# Autonomous division of the outer membrane in Gram-negative bacteria

**DOI:** 10.1101/2025.05.15.654258

**Published:** 2025-05-15

**Authors:** Carolina Basurto De Santiago, Beiyan Nan

**Affiliations:** Department of Biology, Texas A&M University, College Station, Texas, USA

## Abstract

Gram-negative bacteria divide by separating two cell wall layers: peptidoglycan (PG) and the outer membrane (OM). In certain model organisms, the OM is tethered to PG, ensuring it closely follows PG throughout invagination, constriction, and separation. In contrast, *Myxococcus xanthus* exhibits autonomous OM partitioning, occurring only after complete PG fission. However, reinforcing the OM-PG connection by overexpressing endogenous or exogenous tethering proteins synchronizes the constriction and fission of both layers.

The cell wall of Gram-negative bacteria consists of two distinct layers: a peptidoglycan (PG) meshwork and an outer membrane (OM). PG is a single macromolecule of glycan strands crosslinked by short peptides. The OM is an asymmetric bilayer with lipopolysaccharides (LPS) in the outer leaflet and phospholipids in the inner leaflet^1^. While PG plays a major role in maintaining cell integrity and morphology, the OM serves as a selective barrier that protects the cell from harmful substances, including antibiotics^2,3^. In certain bacteria, such as *Escherichia coli*, OMs are load-bearing structures that can exhibit even greater stiffness than PG^4^.

During cell division, bacteria constrict and divide PG using a well-organized PG remodeling machinery called the divisome^5^. In contrast, the OMs in Gram-negative bacteria are devoid of energy sources and consequently lack their own dedicated division machinery. To divide the OM, some Gram-negative bacteria tether their OMs to PG. For instance, *E. coli* employs multiple PG tethering systems for the OM. First, the OM contains abundant Braun’s lipoprotein (Lpp) that attaches to PG directly through covalent bonds^6^. Second, the OM lipoprotein LpoA and LpoB directly interact with PG synthases to coordinate the growth and division of these two cell wall layers^7–10^. Third, the PG-associated lipoprotein (Pal) attaches to PG noncovalently and connects to the OM components in the Tol-Pal system^11^. Fourth, some β-barrel OM proteins (OMPs) interact with PG noncovalently through their C-terminal OmpA-like domains^12^. Through these tethering mechanisms, the OM closely follows the invagination, constriction, and separation of PG throughout *E. coli* cell division^8,13^. However, Lpp and LpoA/B are only conserved in γ-proteobacteria^9^. It is unclear if the coordinated constriction of PG and OM is a universal division mechanism among Gram-negative bacteria and if the connection between OM and divisome is essential for OM division.

To answer these questions, we investigated cell division in *Myxococcus xanthus*, a rod-shaped Gram-negative bacterium. Lacking Lpp and LpoA/B homologs^14^, *M. xanthus* may exemplify a wide group of Gram-negative bacteria that lack rigid connections between the OM and PG. Accordingly, in contrast to the rigid *E. coli* OM, the *M. xanthus* OM appears highly fluid, enabling the rapid diffusion of OM proteins, frequent shedding of OM, and active intercellular exchange of OM contents among closely related strains^15–17^. Using *in situ* cryogenic electron microscopy (cryoEM), we captured the micrographs of dividing *M. xanthus* cells, in which the electron densities of the inner membrane (IM) and OM were easily distinguished from the background (Fig. 1A). In contrast, the thin PG layer was extremely difficult to visualize (Fig. 1A). Since turgor pressure pushes the fluid IM layer against PG, IM closely mirrors the shape of PG during both growth and division. Therefore, we used IM to reflect the constriction and division of PG.

In all CryoEM images of dividing wild-type *M. xanthus* cells, the OM exhibited only slight invagination upon complete IM separation, suggesting that PG and OM are uncoupled during division (Fig. 1A1 – 1A3). This phenotype is strikingly similar to two *E. coli* filamentous mutants that cannot separate daughter cells: the *ΔenvC ΔnlpD* mutant that cannot hydrolyze septal PG^13^ and the *Δlpp Δpal* strain that loses the major connections between PG and OM^18^. However, wild-type *M. xanthus* do not form cell chains, indicating that the uncoupled division of PG and OM does not lead to division defects. To confirm the cryoEM observation, we grew cells in the presence of TAMRA 3-amino-D-alanine (TADA), a red fluorescent D-amino acid that incorporates into PG^19^ and stained the OM with Alexa Fluor 488-conjugated wheat germ agglutinin^20^. We selected dividing cells with cytoplasms separated by fewer than 3 pixels (480 nm) in bright-field images and used fluorescence microscopy to visualize a ~200-nm thick longitudinal section containing both PG and OM under highly inclined and laminated optical sheet (HILO) illumination^21–24^. The fluorescence signals of PG at division sites either intensified or vanished entirely, corresponding to the invaginated and separated PG, respectively (Fig. 1B). Regardless of the separation of PG, all the dividing cells (n > 100) displayed continuous OM at their division sites (Fig. 1B). Crucially, we never detected a surge in OM signal at division sites, confirming that, unlike PG, OM does not undergo gradual invagination in wild-type *M. xanthus* (Fig. 1B). Consistent observations from cryoEM and fluorescence microscopy confirmed that, unlike *E. coli*, *M. xanthus* divides its OM autonomously. Based on our recent discovery that the *M. xanthus* OM is highly fluid^15^, here we propose a straightforward stretch-and-snap mechanism, where the mechanical stretching of the OM drives its division. However, fluorescence microscopy at up to 67 Hz (15 ms/frame) failed to capture such snapping events, suggesting they occurred faster than our detection threshold. Nevertheless, supporting our hypothesis, cryoEM imaging revealed instances of stretched and ruptured OMs between separating daughter cells. (Fig. 1A2, 1A3).

To investigate how PG-tethering mechanisms affect the mode of OM division, we overexpressed *E. coli* Lpp exogenously in *M. xanthus* and observed synchronized invagination, constriction, and constriction of OM and IM (Fig. 1A4, 1A5). Consistently, OM was also synchronized with PG during cell division under fluorescence microscopy (Fig. 1B). Notably, we detected a surge in OM signals at certain division sites, suggesting that OM invaginated in a manner similar to PG (Fig. 1B). In contrast, the overexpression of a truncated Lpp that lacked the terminal lysine (Lppδk) and therefore was unable to form covalent bonds with PG, did not synchronize the division of OM with the IM or PG (Fig. 1A8, 1B). Similar to most Gram-negative bacteria, *M. xanthus* possesses a conserved Tol-Pal system and several OmpA-like OMPs, albeit cryoEM and fluorescence imaging demonstrated that their native expression fails to couple the OM to PG during division (Fig. 1A1 – 1A3). However, similar to the exogenous Lpp, endogenous overexpression of Pal successfully synchronized the invagination, constriction, and separation of OM with both the IM and PG (Fig. 1A6, 1A7, 1B). Thus, reinforcing the OM-PG connection is sufficient to synchronize their division.

This study demonstrates that while Gram-negative bacteria commonly coordinate PG and OM synthesis^7,25^, coupled constriction and fission of these two cell wall layers is not universally conserved for cell division. OM division may occur autonomously through a solely physical mechanism, independent of PG-tethering.

## Supplementary Material

1

## Figures and Tables

**Fig. 1.**
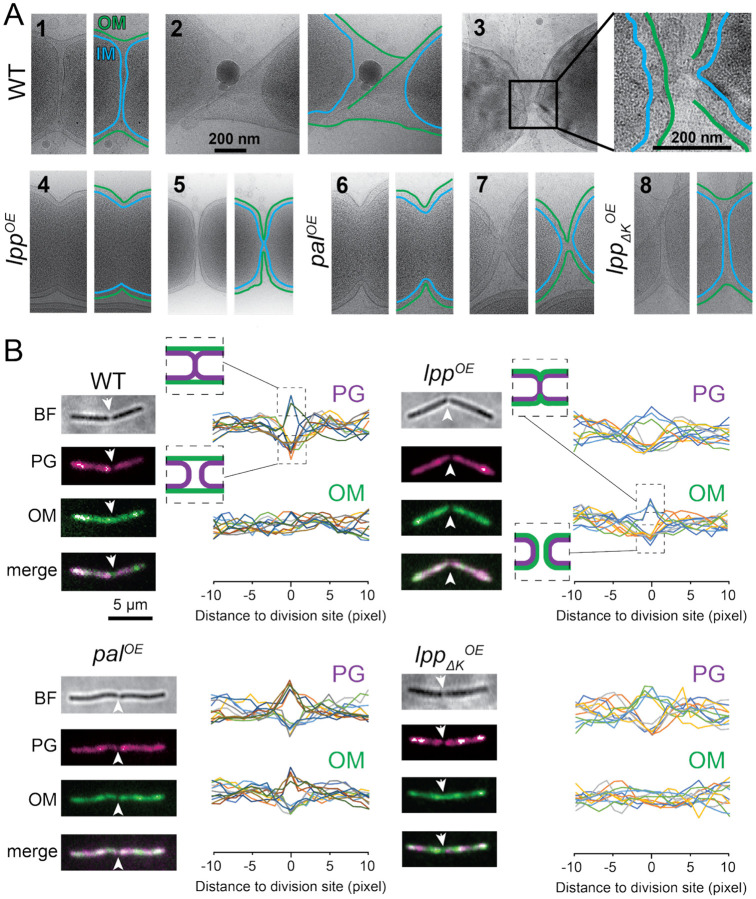
Uncoupled division of PG and the OM in the Gram-negative bacterium *M. xanthus*. **A)** Cryo-EM images of the OMs and IMs at division sites in the wild-type (WT) cells (**1**, the OM remained connected after the division of IM; **2**, the stretched and twisted OM at a division site; **3**, OM fracture when the daughters cells separate), the cells exogenously overexpressing *E. coli* Lpp that attaches the OM to PG (*lpp*^*OE*^, **4** and **5**), the ones overexpressing Pal endogenously (*pal*^*OE*^, **6** and **7**), and the ones exogenously overexpressing a truncated *E. coli* Lpp that cannot attach the OM to PG (*lpp*_*ΔK*_^*OE*^, **8**). **B)** Fluorescence images of the OM and PG in division cells. BF, bright field. White arrows point to the division sites. The fluorescence intensities of PG and OMs from 12 cells are shown for each strain. The identities of cells are distinguished by colors and the fluorescence intensity values of PG and OM were normalized using Z-score standardization.
