# Supplementary material for: Autonomous division of the outer membrane in Gram-negative bacteria
Source: bioRxiv. 2025 May 15:2025.05.15.654258. Preprint. [Version 1] doi: 10.1101/2025.05.15.654258 (PMC12132463; doi:10.1101/2025.05.15.654258)
Supplement: 1 [file NIHPP2025.05.15.654258V1-supplement-1.pdf]

bioRxiv preprint doi: https://doi.org/10.1101/2025.05.15.654258; this version posted May 15, 2025. The copyright holder for this preprint (which was not certified by peer review) is the author/funder, who has granted bioRxiv a license to display the preprint in perpetuity. It is made available under aCC-BY 4.0 International license.

## Materials and methods

Vegetative *M. xanthus* cells were grown in liquid CYE medium (10 mM MOPS pH 7.6, 1% (w/v) Bacto™ casitone (BD Biosciences), 0.5% yeast extract and 8 mM $MgSO_4$) at 32 °C, in 125-ml flasks with vigorous shaking, or on CYE plates that contains 1.5% agar. We used strain DZ2 as the wild-type *M. xanthus* strain [26]. Overexpression mutants were constructed by electroporating DZ2 cells with 4 μg of plasmid DNA. The genes, driven by a vanilate-inducible promoter, were inserted into the Mx4 phage attachment site as merodiploids on the *M. xanthus* chromosome. Transformed cells were plated on CYE plates supplemented with 10 mg/ml tetracycline and expression was induced by 100 μM sodium vanilate. The strains and plasmids used in this study are listed in **Table S1**.

**Table S1. Strains and plasmids used in this study**

| Strain name | Description | Source |
|---|---|---|
| DZ2 | The wild-type strain | [26] |
| BN380 | DZ2 with $P_{van}$-*lpp* | This study |
| BN381 | DZ2 with $P_{van}$-$lpp_{\Delta K}$ | This study |
| BN382 | DZ2 with $P_{van}$-*pal* | This study |

| Plasmid name | Description | Source |
|---|---|---|
| pMR3629 | Plasmid for overexpressing genes as merodiploids using a vanilate-induced promoter, $tet^R$ | [27] |
| pMR3629-lpp | $P_{van}$-*lpp* in pMR3629, $tet^R$ | This study |
| pMR3629-$lpp_{\Delta K}$ | $P_{van}$-$lpp_{\Delta K}$ in pMR3629, $tet^R$ | This study |
| pMR3629-pal | $P_{van}$-*pal* in pMR3629, $tet^R$ | This study |

Bacterial strains were grown overnight in CYE media with appropriate antibiotics, incubated with shaking at 32 °C and 250 rpm to an optical density of $OD_{600}$ 0.6. Cells were

bioRxiv preprint doi: https://doi.org/10.1101/2025.05.15.654258; this version posted May 15, 2025. The copyright holder for this preprint (which was not certified by peer review) is the author/funder, who has granted bioRxiv a license to display the preprint in perpetuity. It is made available under aCC-BY 4.0 International license.

collected by centrifugation (3 min, 6,000 × g, 25 ∘C) and resuspended in CYE media to a final $OD_{600}$ 12. This cell suspension (3 µl) was applied to C Flat-1.2/1.3 200 mesh copper grids (Electron Microscopy Sciences) that were glow discharged for 30 seconds at 15 mA. Grids were plunge-frozen in liquid ethane with an FEI Vitrobot Mark IV (Thermo Fisher Scientific) at 4 °C, 100% humidity with a waiting time of 30 s, two-side blotting time of 2.5 - 3 s, and blotting force of 0. All subsequent grid handling and transfers were performed in liquid nitrogen. Grids were clipped onto cryo-FIB autogrids (Thermo Fisher Scientific).

Images were acquired using a Thermo Fisher Scientific Titan Krios G4 transmission electron microscope, equipped with a Gatan K3 direct electron detector and a Gatan BioContinuum energy filter. Imaging was performed using a 15 eV slit width on the energy filter to enhance image contrast. Micrographs were recorded in counted mode at a nominal magnification of 33,000x, using a dose rate of 14.65 electrons/pixel/second over a 2.6-second exposure, resulting in a total accumulated dose of 50 electrons/$Å^2$.

For fluorescence microscopy, cultures were grown in liquid CYE to $OD_{600}$ ~1 and supplemented with 10 µg/mL wheat germ agglutinin Alexa Fluor-488 conjugate and 75 µM TADA for one hour. Cells were spun down at 6,000x g for 3 min and the pellet washed three times with CYE. 5 µl of cells were spotted on agar (1.5%) pads and imaged using a Andor iXon Ultra 897 EMCCD camera (effective pixel size 160 nm) on an inverted Nikon

bioRxiv preprint doi: https://doi.org/10.1101/2025.05.15.654258; this version posted May 15, 2025. The copyright holder for this preprint (which was not certified by peer review) is the author/funder, who has granted bioRxiv a license to display the preprint in perpetuity. It is made available under aCC-BY 4.0 International license.

Eclipse-Ti™ microscope with a 100× 1.49 NA TIRF objective. Fluorescence of TADA and Alexa Fluor 488-conjugated wheat germ agglutinin was excited by the 561-nm and 488-nm lasers, respectively. Fluorescence intensities were quantified using the ImageJ suite (https://imagej.net) and normalized by Z-score standardization using $Z = (\chi - \mu)/\sigma$, where $Z$, $\chi$, $\mu$, and $\sigma$ are the standard score, observed value, mean, and standard deviation, respectively.
